# AIE-Featured Redox-Sensitive Micelles for Bioimaging and Efficient Anticancer Drug Delivery

**DOI:** 10.3390/ijms231810801

**Published:** 2022-09-16

**Authors:** Wei Zhao, Zixue Li, Na Liang, Jiyang Liu, Pengfei Yan, Shaoping Sun

**Affiliations:** 1 Department of Pharmaceutical Engineering, School of Chemistry and Material Science, Heilongjiang University, Harbin 150080, China; 2 College of Chemistry and Chemical Engineering, Harbin Normal University, Harbin 150025, China

**Keywords:** polymeric micelles, aggregation-induced emission, bioimaging, redox-sensitive, anticancer drug delivery

## Abstract

In the present study, an amphiphilic polymer was prepared by conjugating methoxy poly(ethylene glycol) (mPEG) with tetraphenylethene (TPE) via disulfide bonds (Bi(mPEG-S-S)-TPE). The polymer could self-assemble into micelles and solubilize hydrophobic anticancer drugs such as paclitaxel (PTX) in the core. Combining the effect of TPE, mPEG, and disulfide bonds, the Bi(mPEG-S-S)-TPE micelles exhibited excellent AIE feature, reduced protein adsorption, and redox-sensitive drug release behavior. An in vitro intracellular uptake study demonstrated the great imaging ability and efficient internalization of Bi(mPEG-S-S)-TPE micelles. The excellent anticancer effect and low systemic toxicity were further evidenced by the in vivo anticancer experiment. The Bi(mPEG-S-S)-TPE micelles were promising drug carriers for chemotherapy and bioimaging.

## 1. Introduction

Cancer is one of the most devastating malignant diseases that threaten the health of humans [1]. Although chemotherapy is the predominant approach for cancer therapy, currently, it is far from being satisfactory [2,3]. As well-known, antitumor drugs cannot distinguish tumor cells from normal cells, and they are usually accompanied by serious side effects. Moreover, the poor water solubility of antineoplastic drugs limits their clinical application. To overcome these issues, nanoscale delivery systems have been developed [4].

Self-assembled polymeric micelles are formed from amphiphilic polymers, and they are effective nanocarriers for anticancer drugs [5]. Due to the unique core-shell structure, the micelles could solubilize the hydrophobic drug in the core, thus improve the bioavailability of the drug [6]. Furthermore, micelles can passively and efficiently accumulate in tumor sites via the enhanced permeation and retention (EPR) effect [7]. However, nanoparticles are easily captured by the reticuloendothelial system (RES) and thus cleared from the blood. The modification by methoxy poly(ethylene glycol) (mPEG) is an effective strategy [8]. mPEG is a hydrophilic polymer without toxicity and immunogenicity [9]. It has been widely used to shield nanoparticles from rapid clearance, thus enhancing the opportunity of the system to passively target to the tumor mass via the EPR effect [10].

Once the micelles reach the desired sites, they are expected to effectively and rapidly release the drug there [11,12]. Due to the significant difference in glutathione (GSH) concentration between the physiological microenvironment (approximately 2–10 μM) and the tumor intracellular microenvironment (approximately 2–10 mM), disulfide bonds could be introduced to the nanocarriers to render the system on-demand drug release behavior [13]. Micelles with disulfide bonds in molecules would be relatively stable during the blood circulation, whereas they would disassemble and release the drug in the tumor [14].

To be ideal nanocarriers, the micelles should also be traceable [15]. Although the introduction of traditional fluorescent dyes could make the system visible, they suffered from aggregation-caused quenching effect in aggregated state, which would greatly reduce the fluorescence (FL) intensity and impede the imaging effect [16,17]. The emergence of aggregation-induced emission (AIE) fluorescent molecules opened up a new avenue to overcome this limitation. Fluorogens with AIE characteristics can intensely fluoresce at high concentrations or in aggregated state [18]. The generally accepted mechanism is that the restriction of intramolecular rotations prohibits energy dissipation via nonradiative channels [19,20]. As a typical AIE luminogen, tetraphenylethene (TPE) has attracted wide attention in the field of biological imaging due to its excellent optical properties and easy modification [21,22]. Moreover, TPE could also be used as the hydrophobic segment to form micelles [23,24].

Inspired by the above advantages, in the present study, an amphiphilic AIE-featured polymer was designed and prepared by conjugating mPEG with TPE via disulfide bonds (Bi(mPEG-S-S)-TPE). It is expected that the polymer could self-assemble into micelles with mPEG served as the shell and TPE served as the core. The hydrophobic paclitaxel (PTX) could be solubilized in the core. The introduction of biocompatible mPEG could reduce protein adsorption. TPE would render the system AIE-based imaging property. The disulfide linkage could make the micelles quickly release the drug in the tumor environment with high GSH concentration. The synthesis of Bi(mPEG-S-S)-TPE, fabrication and characterization of PTX-loaded Bi(mPEG-S-S)-TPE micelles, AIE feature of the micelles, and in vitro and in vivo evaluations were investigated in detail.

## 2. Results and Discussion

### 2.1. Synthesis and Characterization of Bi(mPEG-S-S)-TPE

**Scheme 1:**
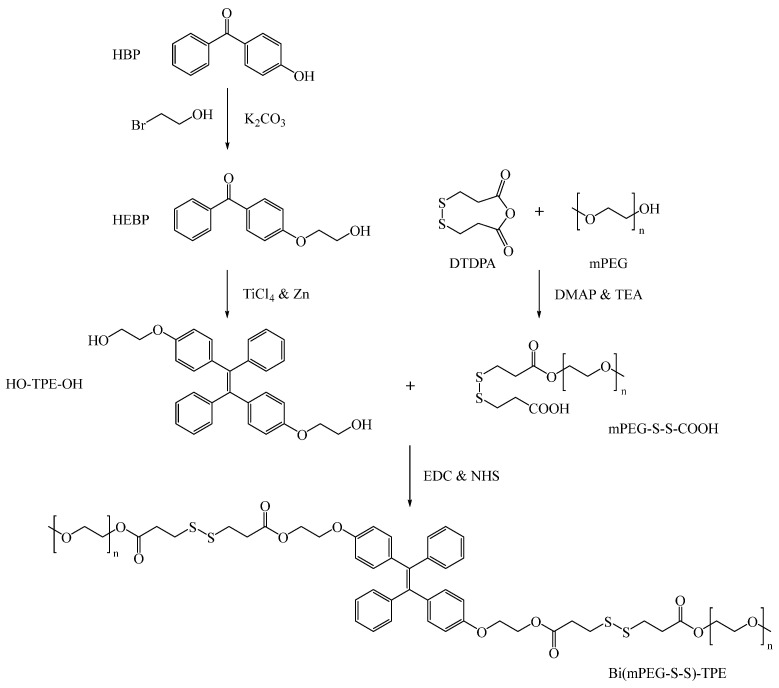
Synthetic route of Bi(mPEG-S-S)-TPE.

The amphiphilic polymer Bi(mPEG-S-S)-TPE, which consisted of AIE-active hydrophobic TPE, disulfide bond, and hydrophilic mPEG, was designed and synthesized by linking two molecules of disulfide bonds modified mPEG (mPEG-S-S-COOH) with dihydroxy modified TPE (HO-TPE-OH). As illustrated in Scheme 1, HO-TPE-OH was prepared by a two-step reaction. Specifically, the reaction between 4-hydroxybenzophenone (HBP) and 2-bromoethanol was used to generate 4-(2-hydroxyethoxy) benzophenone (HEBP) by the Williamson method in the presence of K_2_CO_3_. Then the carbonyl groups of HEBP were coupled through the McMurry coupling reaction, and the alkene HO-TPE-OH was prepared [25]. On the other hand, mPEG-S-S-COOH was obtained through the reaction between the hydroxyl groups of mPEG and anhydride groups of 3,3′-dithiodipropionic anhydride (DTDPA). Finally, the polymer Bi(mPEG-S-S)-TPE was prepared via the esterification of HO-TPE-OH with mPEG-S-S-COOH. The theoretical molecular weight was about 4634 Da.

**Figure 1:**
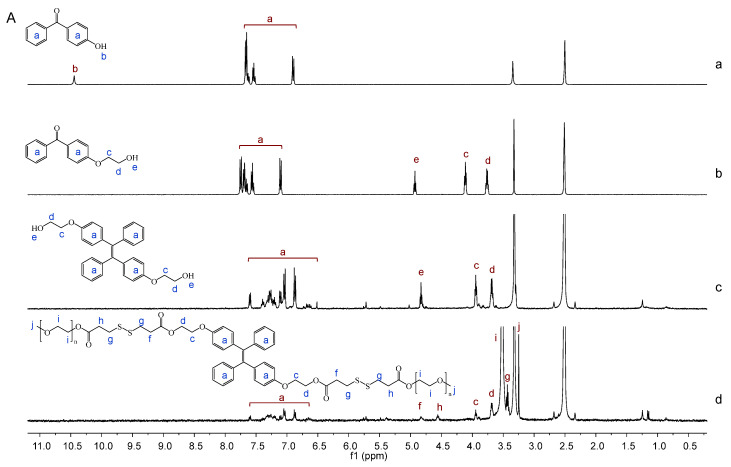

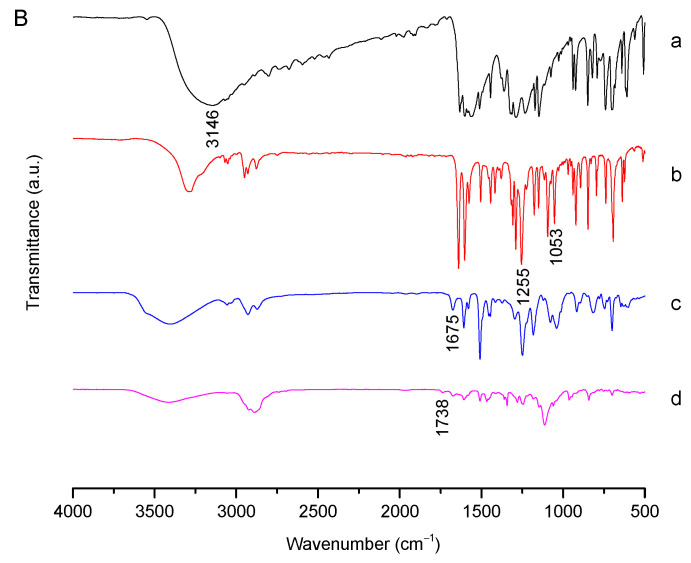
Characterization of Bi(mPEG-S-S)-TPE. (**A**) ^1^H NMR spectra of (**a**) HBP, (**b**) HEBP, (**c**) HO-TPE-OH, and (**d**) Bi(mPEG-S-S)-TPE. (**B**) FT-IR spectra of (**a**) HBP, (**b**) HEBP, (**c**) HO-TPE-OH, and (**d**) Bi(mPEG-S-S)-TPE.

The formation of Bi(mPEG-S-S)-TPE was verified by ^1^H NMR spectra. As presented in Figure 1A, for HBP, signals at 10.45 ppm (H-b) and 6.89–7.67 ppm (H-a) were assigned to the phenolic hydroxyl protons and aromatic protons, respectively. In the spectrum of HEBP, the characteristic signal of phenolic –OH (H-b) disappeared, and signals assigned to the methylene protons (–CH_2_–) from 2-bromoethanol were observed at 4.11 ppm (H-c) and 3.77 ppm (H-d). For HO-TPE-OH, signals at 3.94 ppm (H-c) and 3.69 ppm (H-d) were assigned to the methylene protons (–CH_2_–) of HEBP. Signals at 6.86–7.61 ppm (H-a) were ascribed to the aromatic protons. For Bi(mPEG-S-S)-TPE, the typical signal of methylene protons (–CH_2_–) in mPEG was observed at 3.52 ppm (H-i). Characteristic signals of aromatic rings from HO-TPE-OH were at 6.86–7.61 ppm (H-a). The newly emerged signal at 4.83 ppm (H-f) was due to the formation of the ester bond (–CO–O–CH_2_–) between mPEG-S-S-COOH and HO-TPE-OH. These results demonstrated the successful synthesis of Bi(mPEG-S-S)-TPE.

In addition, FT-IR analysis was also performed to confirm the formation of Bi(mPEG-S-S)-TPE. As shown in Figure 1B, for HBP, the band at 3146 cm^−1^ was due to the O–H stretching vibration of the phenolic hydroxyl group (Ar–OH). For HEBP, bands at 1255 cm^−1^ and 1053 cm^−1^ were due to the stretching vibration of Ar–O and O–CH_3_, respectively. In the spectrum of HO-TPE-OH, the band at 1675 cm^−1^ was ascribed to the stretching vibration of the newly formed ethylenic bond (C=C). For Bi(mPEG-S-S)-TPE, the new band at 1738 cm^−1^ was attributed to the stretching vibration of the generated ester bond (C=O). These pieces of evidence revealed that Bi(mPEG-S-S)-TPE was successfully synthesized.

### 2.2. Determination of Optical Properties

**Figure 2:**
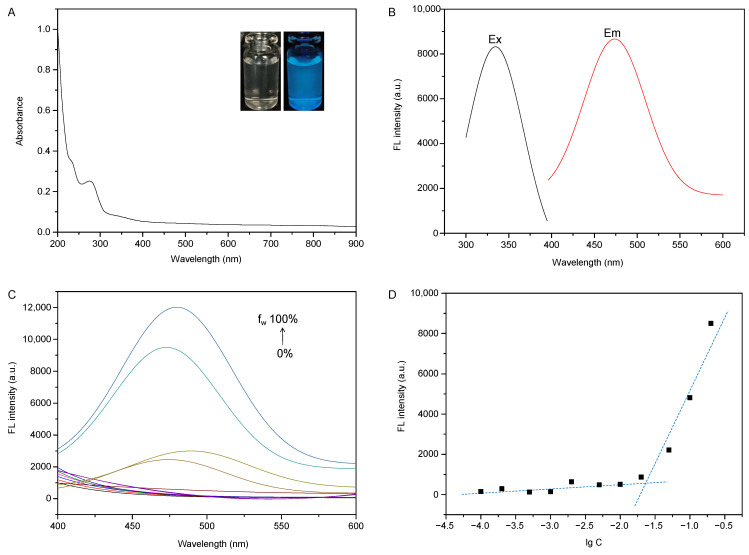
Optical properties of Bi(mPEG-S-S)-TPE. (**A**) UV–Vis spectrum of Bi(mPEG-S-S)-TPE in water. Insets are photographs of Bi(mPEG-S-S)-TPE aggregates in water (left: exposure to a daylight lamp; right: exposure to a UV lamp at 365 nm). (**B**) Excitation and emission spectra of Bi(mPEG-S-S)-TPE in water. (**C**) Fluorescent spectra of Bi(mPEG-S-S)-TPE in THF–water mixtures. (**D**) Relationship between FL intensity and the logarithm of Bi(mPEG-S-S)-TPE concentration.

Bi(mPEG-S-S)-TPE was expected to exhibit excellent fluorescent properties due to the typical AIE effect of TPE. To confirm this inference, the optical property of Bi(mPEG-S-S)-TPE was investigated. From Figure 2A, it was obvious that the maximum absorption wavelength of Bi(mPEG-S-S)-TPE was located at 228 nm, corresponding to the π–π* transition of aromatic rings. In the UV–Vis spectrum, the level-off tails in the visible light region could be attributed to the light scattering of the particles [26], and this phenomenon supported the formation of nanoaggregates in aqueous milieu. Moreover, as evidenced by the insets in Figure 2A, good light transmittance was found for Bi(mPEG-S-S)-TPE dispersion in water. After exposure to UV light at 365 nm, uniform blue fluorescence was observed. It could be deduced that the polymer Bi(mPEG-S-S)-TPE had good water dispersibility, and it was possible to be used for bioimaging application [27,28].

The fluorescent properties of Bi(mPEG-S-S)-TPE were investigated in detail. As illustrated in Figure 2B, the maximum emission wavelength of Bi(mPEG-S-S)-TPE was at 467 nm, and the maximum excitation wavelength was at 336 nm. The large Stokes shift (131 nm) rendered the polymer unique optical advantages, such as minimal self-quenching, enhanced signal fidelity, and high-performance imaging ability [29]. Moreover, the excitation band of Bi(mPEG-S-S)-TPE was relatively broad, which is beneficial for bioimaging. The quantum yield of Bi(mPEG-S-S)-TPE measured using quinine sulfate as the reference was as high as 25.1%, indicating that Bi(mPEG-S-S)-TPE would be suitable for bioimaging application with high imaging efficiency [30].

For TPE display AIE behavior [31], the polymer Bi(mPEG-S-S)-TPE would exhibit an AIE effect in aggregated state. The AIE feature of Bi(mPEG-S-S)-TPE was studied in the mixtures of tetrahydrofuran (THF) and water with different water fractions (f_w_). As illustrated in Figure 2C, the FL intensity of Bi(mPEG-S-S)-TPE was enhanced with the increase in f_w_, especially when f_w_ > 80%. The plausible explanation would be the formation of Bi(mPEG-S-S)-TPE aggregates, which restrict the free movement of Bi(mPEG-S-S)-TPE. More specifically, at low f_w_, Bi(mPEG-S-S)-TPE was dissolved in the solvent, and the molecules could freely rotate. With the addition of water, Bi(mPEG-S-S)-TPE started to form aggregates, and the intramolecular rotation was restricted. As expected, excellent fluorescence was observed.

Critical micelle concentration (CMC) is the lowest concentration of the polymer to form micelles, which is often employed to evaluate the stability of micelles. Due to its amphiphilicity, Bi(mPEG-S-S)-TPE could self-assemble to form micelles when exposed to an aqueous environment. The plot of the FL intensity versus the logarithm of the Bi(mPEG-S-S)-TPE concentration was used to determine the CMC via the tangent method. As presented in Figure 2D, the FL intensity abruptly enhanced at the CMC, which was determined to be 0.024 mg/mL. The low CMC allowed the micelles remain their structure upon high dilution in vivo.

### 2.3. Characterization of PTX-Loaded Bi(mPEG-S-S)-TPE Micelles

The amphiphilicity of Bi(mPEG-S-S)-TPE makes it trend to self-assemble into micelles, with TPE aggregated to serve as the core and mPEG formed the shell. PTX could be loaded into the micelles due to the hydrophobic interaction. For the optimized PTX-loaded micelles, the particle size was 96 ± 3.9 nm, and PDI was 0.358. Notably, such size facilitates the particles to spontaneously aggregate in the tumor via the EPR effect, keep a low level of uptake by the RES, and minimize renal excretion [32]. Zeta potential measurement revealed the PTX-loaded micelles charged negative potential, with a value as high as −39.9 mV, which implied that there was repulsive force among the particles, and the micelles would exhibit excellent stability. Moreover, the negative charge would protect the micelles from recognition by the RES [33]. The drug loading and encapsulation efficiency were determined to be 6.8% and 74.8%, respectively.

**Figure 3:**
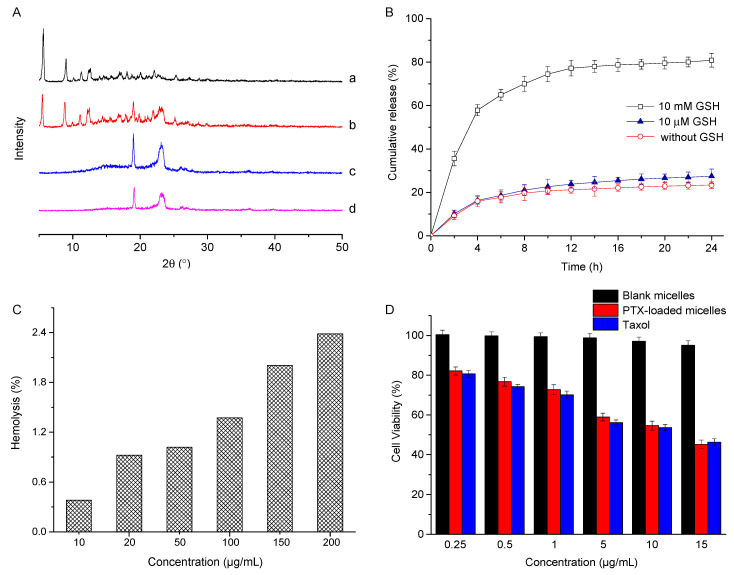
Characterization of PTX-loaded Bi(mPEG-S-S)-TPE micelles. (**A**) XRD diagrams of (**a**) PTX, (**b**) physical mixture of PTX and bare micelles, (**c**) PTX-loaded Bi(mPEG-S-S)-TPE micelles, and (**d**) bare Bi(mPEG-S-S)-TPE micelles. (**B**) In vitro drug release patterns of PTX-loaded Bi(mPEG-S-S)-TPE micelles under different GSH concentrations. (**C**) Hemolysis of PTX-loaded Bi(mPEG-S-S)-TPE micelles at different concentrations. (**D**) In vitro cytotoxicity of PTX-loaded Bi(mPEG-S-S)-TPE micelles against MCF-7 cells.

To confirm the existing state of PTX in Bi(mPEG-S-S)-TPE micelles, XRD analysis was conducted. As observed from Figure 3A, PTX showed diffraction peaks in the 2θ range of 5° to 30°. The pattern of the physical mixture was the simple superposition of the diagrams of blank micelles and PTX. In contrast, the diagram of drug-loaded micelles was similar as the bare micelles without any crystalline peaks of PTX detected, which implied that PTX was loaded in the micelles.

### 2.4. Protein Adsorption Study

As exogenous particles, the micelles were easily cleared by RES. To minimize nonspecific protein adsorption and thus reduce the premature clearance, mPEG was used as a “stealth coating” by serving as the outer shell of the micelles. The effectiveness of mPEG modification was evaluated by a protein adsorption study. From the result, it was obvious that HO-TPE-OH strongly interacted with bovine serum albumin (BSA), and the adsorption value was as high as 30.6%. In comparison, Bi(mPEG-S-S)-TPE showed slight BSA adsorption with a value of 15.9%. It was reasonable to presume that the mPEG shell formed a hydration layer surrounding the micelles [34] and therefore prevented the protein adsorption onto the particles, reduced the rate of clearance, and resulted in prolonged blood circulation time [35,36].

### 2.5. In Vitro Drug Release Study

To confirm the redox-sensitive property of Bi(mPEG-S-S)-TPE micelles, their drug release behavior was investigated in PBS 7.4 with different GSH concentrations. As presented in Figure 3B, the amount of PTX released in the medium without GSH was 23.4% within 24 h, and the value was about 27.5% for 10 μM GSH. In contrast, when GSH concentration increased to 10 mM, the value reached about 80.9%. The faster drug release at high GSH concentration could be due to the disassembly of micelles by the disruption of redox-sensitive disulfide bonds. In other words, the disulfide bond endowed the Bi(mPEG-S-S)-TPE micelles with GSH responsiveness. It could be inferred that the micelles would specifically release the drug at tumor sites, and the antitumor efficacy would be enhanced.

### 2.6. Hemolysis Assay

The hemocompatibility evaluation is substantial for drug carriers that would be applied via intravenous injection [37]. As obtained from a hemolysis study (Figure 3C), the hemolysis ratio of drug-loaded Bi(mPEG-S-S)-TPE micelles exhibited a dose-dependent increase. Even if the concentration was as high as 200 μg/mL, the hemolysis rate was only 2.4%. Considering that the micelles would be highly diluted when administered in vivo, it could be inferred that the Bi(mPEG-S-S)-TPE micelles were relatively safe without inducing any noticeable damage to erythrocytes.

### 2.7. Cytotoxicity Evaluation

The ability of PTX-loaded Bi(mPEG-S-S)-TPE micelles to inhibit tumor cell growth was evaluated by the MTT method using Taxol as the control. As presented in Figure 3D, the viability of MCF-7 cells treated with blank micelles was above 95% at all the investigated levels, which indicated the biocompatibility and nontoxicity of the polymer. It was also apparent that the PTX-loaded micelles caused a concentration-dependent growth inhibition effect on MCF-7 cells, and the cytotoxicity was comparable with that of Taxol at the same concentration. This could be due to the quick release of the drug in the reducing microenvironment of cancerous cells, which has been proved in the drug release study. The Bi(mPEG-S-S)-TPE micelles would be potential carriers for antitumor drugs.

### 2.8. Cellular Uptake and Cellular Imaging Behavior Study

**Figure 4:**
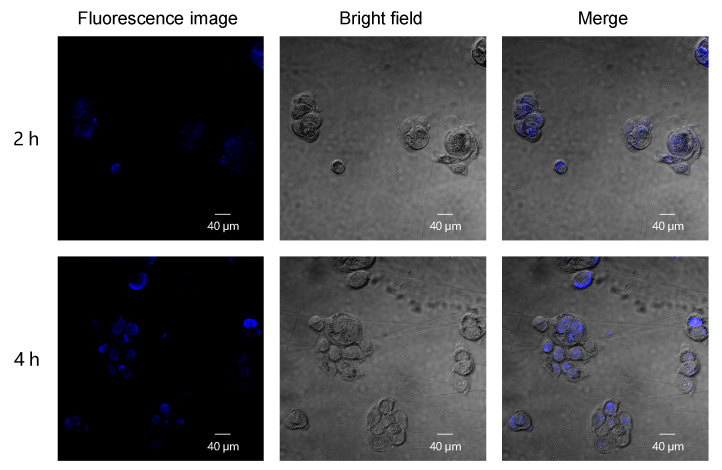
Cellular internalization of Bi(mPEG-S-S)-TPE micelles by MCF-7 cells.

The bioimaging potential and cellular internalization behavior of Bi(mPEG-S-S)-TPE micelles were monitored by a confocal laser scanning microscope (CLSM). As presented in Figure 4, strong blue fluorescence was observed in the cytoplasm of cells after the cells were incubated with Bi(mPEG-S-S)-TPE micelles for 2 h. Moreover, the fluorescence became brighter when the incubation time increased to 4 h. The mean fluorescence intensity (MFI) at 2 h and 4 h was 70.4 ± 5.9 and 80.6 ± 4.8, respectively, which quantitatively indicated the efficient internalization of Bi(mPEG-S-S)-TPE micelles and their incubation time-dependent cellular uptake behavior. On the other hand, these results also revealed that the Bi(mPEG-S-S)-TPE micelles possessed desirable AIE characteristic, and they could track themselves in cancer cells. The Bi(mPEG-S-S)-TPE micelles possessed great potential for bioimaging and antitumor drug delivery applications.

### 2.9. In Vivo Antitumor Activity Study

**Figure 5:**
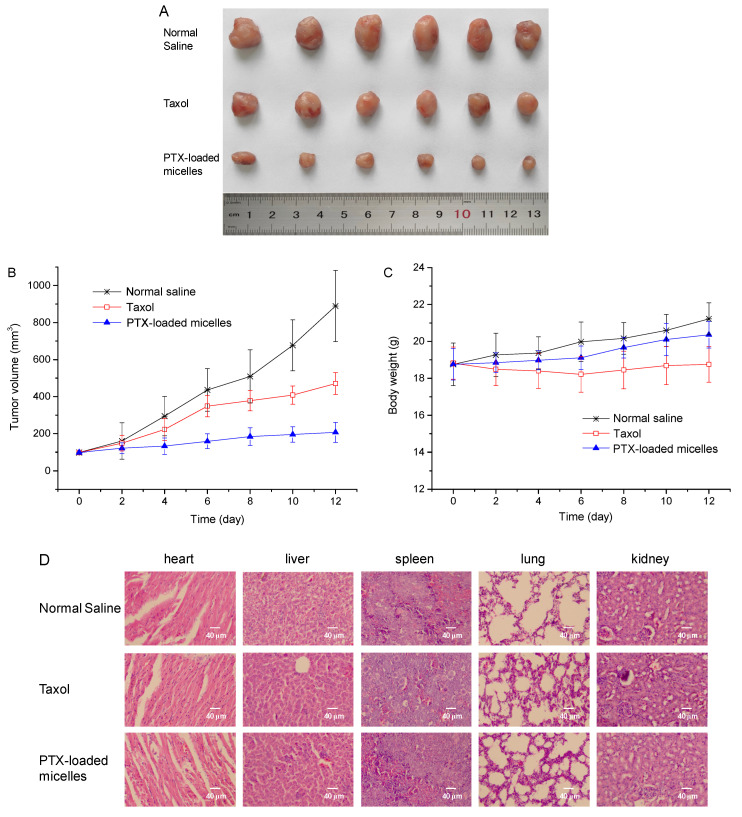
In vivo tumor growth suppression effect and systemic toxicity of PTX-loaded Bi(mPEG-S-S)-TPE micelles in 4T1-tumor bearing mice. (**A**) Photo of tumors from different formulation-treated groups. (**B**) Tumor volume curves of mice in different treatment groups. (**C**) Body weight curves of mice in different treatment groups. (**D**) Histological analysis of major organs in different treatment groups.

4T1 tumor-bearing BALB/c mice were employed to assess the tumor growth suppression effect of the PTX-loaded Bi(mPEG-S-S)-TPE micelles. As presented in Figure 5A, in comparison with the normal saline group, both treatment groups exhibited significant tumor regression with a much smaller tumor size. In addition, the PTX-loaded Bi(mPEG-S-S)-TPE micelles displayed superior tumor inhibition effect compared with Taxol. The average tumor volume of each group presented in Figure 5B also confirmed the improved antitumor effect of PTX-loaded micelles, which could be ascribed to the advantages of the delivery system. Specifically speaking, the size of no more than 200 nm was beneficial for the micelles to accumulate in the tumor through the EPR effect. The modification by mPEG and the negative charge could prolong the circulation time of micelles and thus strengthen the EPR effect. After entering the tumor tissue, with disulfide linkage in the structure, micelles could rapidly disassemble and release PTX under the action of high concentration of GSH. Taken together, the Bi(mPEG-S-S)-TPE micelles could exhibit high tumor inhibition effect.

To evaluate the potential systemic toxicity of the PTX-loaded micelles, changes in the body weight were monitored. As illustrated in Figure 5C, the mice slightly lost their weight in the Taxol group. In contrast, there was an increasing trend in the body weight of mice in the micelles group, which reflected the low systemic toxicity of the micellar system. This could be due to the specific PTX release in tumor and the reduced PTX exposure to normal tissues.

To further assess the toxicity of PTX-loaded Bi(mPEG-S-S)-TPE micelles, histological analysis was conducted. As shown in Figure 5D, similar to the saline group, the micelles did not induce any obvious damage to the organs of mice. The above results revealed that the Bi(mPEG-S-S)-TPE micelles possessed improved antitumor efficiency without obvious toxicity, and they might be promising carriers for antitumor dug delivery.

## 3. Materials and Methods

### 3.1. Materials

1-Ethyl-3-(3-dimethylaminopropyl) carbodiimide hydrochloride (EDC), N-hydroxysuccinimide (NHS), 4-(dimethylamino) pyridine (DMAP), methoxy poly(ethylene glycol) (mPEG, Mw = 1900 Da), 4-hydroxybenzophenone (HBP), and 2-bromoethanol were obtained from Aladdin Industrial Co., Shanghai, China. 3-(4,5-Dimethylthiazol-2-yl)-2,5-diphenyl tetrazolium bromide (MTT) was purchased from Sigma Chemical Co., St. Louis, MO, USA. Dulbecco’s modified Eagle’s medium (DMEM) and fetal bovine serum (FBS) were obtained from Beyotime Biotechnology Ltd., Shanghai, China.

### 3.2. Cells and Animals

4T1 and MCF-7 cell lines were kindly provided by Harbin Medical University. MCF-7 cells were cultured in DMEM supplemented with 100 IU/mL of penicillin, 10% (*v*/*v*) FBS, and 100 μg/mL of streptomycin. 4T1 cells were cultured in an RPMI 1640 medium supplemented with 10% (*v*/*v*) FBS. Cells were incubated at 37 °C with 5% CO_2_ in a humidified incubator.

BALB/c mice weighing 18–22 g (female mice) were obtained from the Laboratory Animal Center of Harbin Medical University, Harbin, China. Mice were kept under standard conditions.

### 3.3. Synthesis of Bi(mPEG-S-S)-TPE

#### 3.3.1. Synthesis of HO-TPE-OH

The HO-TPE-OH was prepared as previously reported [38]. At first, HEBP was synthesized. HBP (1.9 g), K_2_CO_3_ (2.76 g), and 2-bromoethanol (1.25 g) were mixed, and the reaction was proceeded for 24 h at 110 °C. The resultant was extracted with dichloromethane and then purified by silica gel column chromatography to provide HEBP as a white crystalline solid.

Then HEBP (484 mg), zinc powder (160 mg), THF (3 mL), and TiCl_4_ (0.5 mL) were mixed under nitrogen atmosphere. The reaction was carried out under stirring for 12 h and then quenched by the addition of hydrochloric acid. The resultant was extracted with ethyl acetate and dried over anhydrous Na_2_SO_4_. The crude product was purified by silica gel column chromatography. HO-TPE-OH was isolated as a yellow solid.

#### 3.3.2. Synthesis of mPEG-S-S-COOH

The conjugate mPEG-S-S-COOH was prepared by linking DTDPA with mPEG [39]. At first, DTDPA was synthesized as per a previous report [40]. Then DTDPA (96 mg), mPEG (500 mg), triethylamine (TEA, 70 μL), and 4-dimethylaminopyridine (DMAP, 61 mg) were added to N,N-dimethylformamide (DMF, 10 mL). The mixture was stirred at 35 °C. Twenty-four hours later, iced ethyl ether was added to precipitate the crude product. After solvent removal and further vacuum-drying, mPEG-S-S-COOH was isolated.

#### 3.3.3. Synthesis of Bi(mPEG-S-S)-TPE

Bi(mPEG-S-S)-TPE was synthesized via the esterification between mPEG-S-S-COOH and HO-TPE-OH. Briefly, mPEG-S-S-COOH (350 mg), NHS (55.2 mg), and EDC (92 mg) were added to DMF (10 mL). The reaction was carried out at room temperature for 24 h. Then HO-TPE-OH (37.5 mg) was added, and the reaction was continued for 48 h in the dark. After dialysis against distilled water for 48 h (MWCO: 3500 Da), the product Bi(mPEG-S-S)-TPE was collected by lyophilization.

### 3.4. Characterization of Bi(mPEG-S-S)-TPE

#### 3.4.1. ^1^H NMR and FT-IR Analysis

The structure of Bi(mPEG-S-S)-TPE was analyzed by both ^1^H NMR analysis (AV-400, Bruker, Switzerland) and FT-IR analysis (Tensor II, Bruker, Switzerland).

#### 3.4.2. AIE Behavior Investigation

The excitation and emission spectra of Bi(mPEG-S-S)-TPE were investigated by a fluorescence spectrometer (F-2500 FL, Hitachi Ltd., Tokyo, Japan). A series of THF–water mixtures with different f_w_ were used to determine the AIE behavior of Bi(mPEG-S-S)-TPE. The quantum yield was determined using quinine sulfate as the reference (φ = 0.54 in 0.1 M H_2_SO_4_), and the equation was as follows:

φ_s_ = φ_r_ (A_r_·I_s_·η_s_^2^)/(A_s_·I_r_·η_r_^2^) (1)

where φ refers to the quantum yield; and A, I, and η represent the absorbance intensity, integrated FL intensity, and refractive index of the solvent, respectively. The subscripts s and r represent the sample and reference, respectively.

#### 3.4.3. CMC Measurement

The CMC value of Bi(mPEG-S-S)-TPE was obtained through measuring the change of FL intensity at different concentrations. A series of Bi(mPEG-S-S)-TPE in water dispersions were prepared, with the concentration ranging from 5.0 × 10^−4^ to 0.2 mg/mL. The FL intensity at the emission wavelength of 467 nm was recorded under an excitation of 336 nm.

### 3.5. Preparation and Characterization of Bi(mPEG-S-S)-TPE Micelles

The drug-loaded Bi(mPEG-S-S)-TPE micelles were obtained as follows. At first, the Bi(mPEG-S-S)-TPE dispersion in distilled water was prepared. Then, the PTX in the acetone solution was added. After being sonicated for 3 min (on for 3 s and off for 2 s), the mixture was dialyzed (MWCO of 3500 Da) against distilled water for 2 h and further filtrated with a 0.45 μm filter to obtain the drug-loaded micelles. The micelles could be further freeze-dried and stored as a white powder.

XRD analysis was used to analyze the crystalline characteristic of PTX in the micelles (Geigerflex, Rigaku Co., Tokyo, Japan). Data were collected from 5° to 50° with a step-scan mode. The dynamic light-scattering method was employed to determine the particle size and zeta potential of PTX-loaded micelles (Zetasizer Nano-ZS90, Malvern Instruments, Malvern, UK). The drug loading (DL) and encapsulation efficiency (EE) were calculated using Equations (2) and (3), respectively.

DL(%) = (weight of drug in micelles/weight of drug-loaded micelles) × 100%(2)

EE(%) = (weight of drug in micelles/weight of drug fed initially) × 100%(3)

### 3.6. Protein Adsorption Test

The effect of mPEG on protein adsorption was evaluated using BSA as the model protein. Micelles (0.25 mg/mL) were incubated with the BSA in PBS 7.4 solution (2 mg/mL) at 37 °C under vigorous shaking at 150 rpm for 5 h. After centrifugation at 16,000 rpm for 20 min, the supernatant was collected and analyzed for the concentration of BSA by measuring the absorbance at 278 nm using the standard curve of BSA.

### 3.7. In Vitro Drug Release Study

The GSH responsiveness of PTX-loaded Bi(mPEG-S-S)-TPE micelles was evaluated by the dialysis method in PBS 7.4, which contained 0.5% Tween-80. To simulate a different environment, the GSH concentration in the media was 0 μM, 10 μM, and 10 mM, respectively. Samples were incubated in a thermostatic water bath with shaking at 100 rpm. After incubation for different times, 5 mL of the release medium was withdrawn and replenished with the same volume of a blank medium. The PTX content of the sample was quantified.

### 3.8. Hemolysis Assay

The disruption effect of the micelles on the erythrocyte membrane was evaluated [41]. For hemolysis assay, fresh rabbit whole blood was collected, and the erythrocytes were isolated. Then a 2% erythrocytes suspension was prepared with physiological saline. Different amounts of PTX-loaded micelles were mixed with the erythrocytes suspension, and a series of samples were obtained. All samples were incubated in a 37 °C water bath for 2 h. After centrifugation at 3000 rpm for 10 min, the absorbance of the supernatant was determined at 540 nm (UV mini-1240, Shimadzu, Japan). According to Equation (4), the hemolysis level was calculated.

Hemolysis (%) = (A_sample_ − A_0%_)/(A_100%_ − A_0%_) × 100% (4)

where A_0%_, A_100%_, and A_sample_ are the absorbance of physiological saline, distilled water, and sample, respectively.

### 3.9. In Vitro Cytotoxicity Assay

The cell growth inhibition effect of PTX-loaded Bi(mPEG-S-S)-TPE micelles was determined using the MTT method. Briefly, MCF-7 cells were inoculated in a 96-well plate (1 × 10^4^ cells/well). After incubation at 37 °C for 24 h, cells were treated with the micelles for 24 h. After the addition of an MTT reagent (5 mg/mL), further incubation was continued for 4 h. Then the supernatant was removed, and 0.2 mL of DMSO was added. The absorbance was measured at 490 nm with a Bio-Rad 680 microplate reader (Bio-Rad Laboratories, Hercules, CA, USA). Taxol with the same PTX concentration was used for comparison. The cell viability was obtained according to the following formula:

Cell viability (%) = A_sample_/A_untreated cells_ × 100% (5)

### 3.10. Cellular Uptake and Cellular Imaging Study

The cellular uptake behavior and AIE-active cellular imaging capability of Bi(mPEG-S-S)-TPE micelles were investigated using MCF-7 cells. Cells were cultured in 6-well plates (2 × 10^5^ cells/well) for 24 h. Then Bi(mPEG-S-S)-TPE micelles were added, and the incubation was continued for 2 h and 4 h, respectively. After washing with PBS thrice, cells were observed with a confocal laser scanning microscope (LSM 710, Zeiss, Germany) at the excitation wavelength of 405 nm. Image J software was utilized to quantify the fluorescence intensity.

### 3.11. In Vivo Antitumor Efficacy Study

4T1 cells were subcutaneously inoculated into the mice to establish the tumor xenograft model. Once the tumor reached about 100 mm^3^, treatments were started. The mice were randomly divided into 3 groups (n = 6). Normal saline (the negative control), Taxol (the positive control, PTX of 10 mg/kg) or PTX-loaded Bi(mPEG-S-S)-TPE micelles (PTX of 10 mg/kg) were administered by the tail vein injection at 3-day intervals for 4 times. The body weight and tumor size of each mice were measured every other day. Formula (6) was used to calculate the tumor volume (V). The mice were sacrificed after treatment. Tumors were collected and photographed.

V = 0.5 × length × width^2^ (6)

Histopathological examination was also performed to assess the systemic toxicity of the micelles. Main organs were isolated, fixed with 10% formalin and embedded in paraffin. Then samples were sliced, deparaffinized, and further stained with hematoxylin and eosin (H&E). Observation was conducted by a light microscope (DMC5400, Leica Microsystems Ltd., Heerbrugg, Switzerland).

### 3.12. Statistical Analysis

All experiments were performed at least three times, and values were expressed as mean ± standard deviation (SD). Intergroup difference was assessed for significance using one-way analysis of variance (ANOVA), and *p* < 0.05 was considered statistically significant.

## 4. Conclusions

In this study, an amphiphilic AIE-featured polymer Bi(mPEG-S-S)-TPE was designed and prepared by conjugating mPEG with TPE via disulfide bonds. The polymer was used as micelles for the delivery of paclitaxel. As expected, the Bi(mPEG-S-S)-TPE micelles exhibited excellent AIE properties and reduced protein adsorption and redox-sensitive drug release behavior. The in vitro intracellular uptake study demonstrated the great imaging ability and efficient internalization of Bi(mPEG-S-S)-TPE micelles. The excellent anticancer effect and low systemic toxicity were further evidenced by the in vivo anticancer experiment. All the evidence suggested that the Bi(mPEG-S-S)-TPE micelles were promising drug carriers for chemotherapy and bioimaging.

## Data Availability

Data are contained within the article.
